# Case Report: A “visceral vasculitis storm” in Systemic Lupus Erythematosus: simultaneous enteritis, ureteritis, and splenic infarction in a pediatric patient

**DOI:** 10.3389/fmed.2026.1882588

**Published:** 2026-07-09

**Authors:** Xia Wu, Yan Liu, Huan He

**Affiliations:** Department of Rheumatology and Immunology, Suining Central Hospital, Suining, China

**Keywords:** lupus enteritis, lupus ureteritis, Systemic Lupus Erythematosus, telitacicept, visceral vasculitis

## Abstract

**Background:**

Systemic Lupus Erythematosus (SLE) is a multi-system autoimmune disease. Although lupus enteritis is a recognized manifestation of mesenteric vasculitis, lupus ureteritis is rare. The simultaneous occurrence of both, termed a “visceral vasculitis storm,” poses a significant diagnostic challenge. Clinicians often struggle to differentiate primary ureteritis from secondary mechanical compression caused by intestinal edema (the “monistic” view).

**Case presentation:**

A 16-year-old girl with a history of transient ptosis and positive ANA/dsDNA results 4 months prior to the current presentation, presented with abdominal pain, fever, and transient dysuria. Initial outpatient CT revealed bilateral hydroureteronephrosis. During hospitalization, the patient's condition deteriorated despite antibiotic therapy. A repeat contrast-enhanced CT (CECT) demonstrated the classic “target sign” in the bowel wall, splenic infarction, and crucially, distinct ureteral wall enhancement. Laboratory findings showed a “high CRP/low PCT” dissociation and a markedly elevated D-Dimer (13.74 μg/mL). The diagnosis was revised to SLE with diffuse visceral vasculitis. The patient responded rapidly to methylprednisolone pulse therapy and IVIG, followed by maintenance therapy with Cyclophosphamide and the dual BLyS/APRIL inhibitor Telitacicept.

**Conclusion:**

This case highlights that ureteral wall enhancement on CECT can serve as a potentially useful radiologic clue for primary lupus ureteritis. Recognizing the “visceral vasculitis storm” is essential to avoid the pitfalls of diagnostic monism and to initiate aggressive immunosuppressive therapy promptly to prevent irreversible organ damage.

## 1 Introduction

Systemic Lupus Erythematosus (SLE) is a heterogeneous autoimmune disease characterized by the production of autoantibodies and immune complex deposition, and is capable of affecting virtually any organ system (1, 2). While mucocutaneous (e.g., malar rash) and musculoskeletal (e.g., arthritis) manifestations are the most common presenting features, visceral involvement, specifically of the gastrointestinal (GI) and urinary tracts, represents a severe and potentially life-threatening subset of the disease (3).

“Lupus Enteritis,” primarily caused by mesenteric vasculitis, is recognized as a distinct clinical entity, accounting for approximately 0.2%−5.8% of SLE cases (4–6). It typically presents with abdominal pain, vomiting, and diarrhea. In contrast, “Lupus Ureteritis” is an exceedingly rare manifestation, with fewer than 50 cases reported in the English literature to date (7, 8). It is often overshadowed by the more common “Lupus Cystitis” (interstitial cystitis) or misdiagnosed as secondary hydronephrosis due to mechanical obstruction (9).

The simultaneous occurrence of lupus enteritis and ureteritis is clinically complex. When a patient presents with both intestinal edema and hydronephrosis, the diagnostic challenge lies in distinguishing whether urinary tract involvement is a secondary bystander effect (mechanical compression by the edematous bowel) or primary concurrent vasculitis. This distinction is not merely academic, but fundamentally dictates the therapeutic urgency and prognosis of irreversible fibrosis.

We report a rare and complex case involving a 16-year-old girl. Her clinical course was unique for two reasons: firstly, she exhibited a prodromal neurological sign (transient ptosis) months before the systemic flare; and secondly, she developed a “visceral vasculitis storm” involving the bowel, ureters, and spleen simultaneously. We specifically discuss the utility of ureteral wall enhancement on Contrast-Enhanced Computed Tomography (CECT) as a potentially useful radiologic clue in establishing the diagnosis of primary lupus ureteritis, challenging the traditional “monistic” view of compressive obstruction.

## 2 Case description

### 2.1 Patient information and past medical history

A 16-year-old girl presented with a complex clinical history. The clinical course and management timeline are summarized in Figure 1. Four months prior to the current presentation, she was admitted to the Department of Neurology at our hospital due to right upper eyelid ptosis. Differential diagnoses included Myasthenia Gravis (MG) and Connective Tissue Disease (CTD) (10). Immunological screening revealed strongly positive ANA (1:1,000) and anti-dsDNA antibodies. However, the definitive clinical diagnosis of SLE was delayed, and systemic treatment was not initiated because the patient lacked typical mucocutaneous stigmata such as malar rash or oral ulcers.

**Figure 1:**
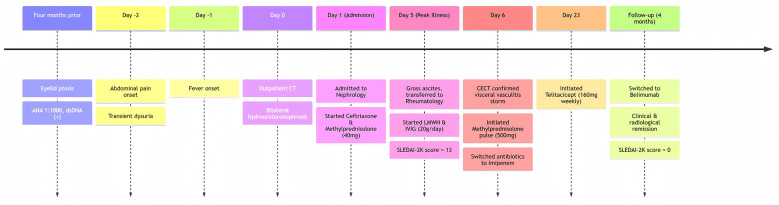
Graphical timeline of clinical events and therapeutic interventions. The timeline illustrates the chronological sequence of the patient's symptoms, diagnostic milestones, and pharmacological treatments relative to the day of admission. SLEDAI-2K: Systemic Lupus Erythematosus Disease Activity Index 2000; LMWH: low-molecular-weight heparin; IVIG: intravenous immunoglobulin; CECT: contrast-enhanced computed tomography.

### 2.2 Acute presentation (nephrology department)

The patient was admitted to the Department of Nephrology on Day 1.

History of Present Illness: One day before admission, the patient developed lower abdominal pain, nausea, vomiting, chills, and fever (undocumented specific temperature). Notably, she reported transient difficulty voiding (dysuria) starting 2 days before admission, which resolved by the time of hospitalization.

Physical Examination on Admission: Physical examination revealed that the patient was alert and oriented. Abdominal examination revealed tenderness, specifically in the lower left and right quadrants. The costovertebral angle (CVA) tenderness (kidney percussion pain) was positive bilaterally. Eyelid ptosis was not observed upon admission.

Laboratory Findings: Blood tests revealed pancytopenia (WBC 3.9 × 10^9^/L, Hb 113 g/L, PLT 142 × 10^9^/L) and an elevated CRP level (12.95 mg/L), paired with a low procalcitonin level (PCT 0.12 ng/ml). Immunological screening showed strongly positive results for antinuclear antibody (ANA, 1:1,000), anti-dsDNA, and anti-Sm antibodies, along with profound hypocomplementemia (C3: 0.48 g/L).

Extensive workups were performed to rule out infectious etiologies. Bilateral blood cultures (four bottles in total), ascites fluid culture, and stool cultures all yielded no bacterial growth. Urinalysis showed no white blood cells or pus cells. Although viral screenings for cytomegalovirus (CMV) and Epstein-Barr virus (EBV) were omitted due to patient refusal at the time, the rapid and sustained resolution of symptoms following high-dose immunosuppression—without antiviral or extended antibiotic escalation—effectively excluded active severe infection.

### 2.3 Diagnostic assessment

Given the fever and abdominal signs in the context of suspected autoimmunity, abdominal infection (e.g., spontaneous bacterial peritonitis) was initially considered. The patient was initiated on empirical antibiotic therapy with Ceftriaxone (0.5 g) combined with low-dose methylprednisolone (40 mg).

### 2.4 Clinical deterioration and transfer

Despite 4 days of antibiotic and low-dose steroid treatment, the patient's condition deteriorated. The abdominal pain recurred and became progressively more severe, accompanied by worsening abdominal distension. On Day 5, she was transferred to the Department of Rheumatology and Immunology.

Physical Examination on Transfer (Day 5): The patient appeared acutely ill. The abdomen was grossly distended (bulging). Tenderness persisted in the lower quadrants and CVA tenderness remained positive. Crucially, shifting dullness was positive, indicating a significant increase in the ascites volume. At this peak of the disease flare (Day 5 of admission), the patient exhibited severe disease activity with a Systemic Lupus Erythematosus Disease Activity Index 2000 (SLEDAI-2K) score of 13.

### 2.5 Radiographic progression

Serial scans revealed rapid progression of the visceral pathology.

On Day 0 (Outpatient): A pelvic CT scan performed at a local hospital revealed mild bilateral hydroureteronephrosis with periureteral exudation preceding the peak of her gastrointestinal symptoms.

On Day 2: Initial CECT showed edema and thickening of the duodenal and jejunal walls with infiltration of the perirenal fascia, but no significant ureteral dilation.

On Day 6 (Peak Illness): Due to worsening abdominal distension, a repeat CECT was performed, revealing a “triple hit” of visceral involvement (11, 12). Specifically, the scan demonstrated prominent edema and muscular layer thickening of the gastric antrum wall (Figure 2A). This was accompanied by a classic “target sign” in the duodenal bulb, along with an irregular luminal caliber and peri-vascular flocculent exudation of the artery along the greater curvature of the stomach, indicative of active vasculitis (Figure 2B) (13). Furthermore, distinct mural enhancement and peri-ureteral flocculent exudation of the bilateral ureters were clearly captured (Figure 2C) (9).

**Figure 2:**
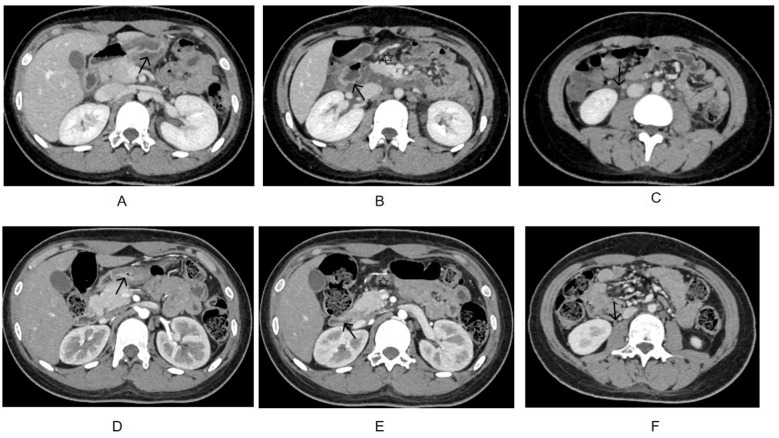
Paired before-and-after contrast-enhanced computed tomography (CECT, portal venous phase, axial view) images demonstrating the resolution of the “visceral vasculitis storm.” **(A–C)** Images obtained during the peak illness show severe multi-organ involvement. **(A)** Prominent edema and muscular layer thickening of the gastric antrum wall (thin arrow). **(B)** The artery along the greater curvature of the stomach demonstrates an irregular luminal caliber and peri-vascular flocculent exudation (star), indicative of active vasculitis. The thin arrow points to the classic “target sign” in the duodenal bulb. **(C)** Peri-ureteral flocculent exudation and distinct mural enhancement of the right ureter (thin arrow) confirm primary intrinsic inflammation. **(D–F)** Corresponding follow-up images obtained at the exact same anatomical levels show dramatic resolution following intensive immunosuppressive therapy. **(D)** Significant resolution of the gastric antrum edema (thin arrow). **(E)** The artery along the greater curvature now shows a uniform caliber with marked absorption of the surrounding exudation (star). The duodenal “target sign” has disappeared (thin arrow). **(F)** Complete resolution of the peri-ureteral exudation and ureteral wall enhancement (thin arrow), objectively corroborating the remission of the visceral vasculitis.

### 2.6 Diagnostic interventions and management of complications

Paracentesis (Day 6): Due to severe distension and massive ascites revealed by ultrasound, a bedside ultrasound-guided paracentesis was performed to relieve pressure and investigate the etiology. Fluid analysis revealed an exudate (high protein/cellularity) consistent with inflammation. However, bacterial culture results were negative, pushing the diagnosis away from septic peritonitis and sterile autoimmune serositis.

Antibiotic Adjustment (Day 9): On Day 8, liver function tests revealed elevated transaminase levels. Considering the potential drug-induced liver injury (DILI) from Ceftriaxone, and to broaden the coverage of potentially resistant organisms in this critical state, the antibiotic regimen was switched to Imipenem/Cilastatin (1.0 g q8h) on Day 9.

Rationale for Dual Therapy: Although antibiotics were maintained to cover possible secondary infection (a common comorbidity in active SLE), the clinical picture—specifically the failure of initial antibiotics to halt symptom progression and the characteristic “target sign” on CT—suggested that abdominal infection could not fully explain the severity of the findings. The primary pathology was identified as active lupus mesenteric vasculitis and ureteritis.

### 2.7 Therapeutic intervention, follow-up, and outcomes

Upon transfer, the treatment focus shifted to aggressive immunosuppression, targeting vasculitis.

Regimen: The patient received IVIG (20 g/day) and Methylprednisolone Pulse Therapy (500 mg/day) (14). Following the acute rescue with pulse methylprednisolone (500 mg/day for 5 days, initiated on Day 6) and IVIG (20 g/day for 3 days, initiated on Day 5), dramatic clinical improvement was observed. Abdominal pain and distension resolved significantly, confirming the autoimmune nature of the crisis. Subsequently, targeted maintenance therapy was explored. On Day 23, the patient was started on telitacicept (160 mg weekly) to rapidly target B-cell hyperactivation (15). However, due to health insurance coverage limitations and financial constraints, the biologic regimen was switched to belimumab (480 mg intravenously) 4 weeks post-discharge (16).

At the latest follow-up, approximately 4 months post-discharge, the patient remained in complete clinical and serological remission. She was asymptomatic with a SLEDAI-2K score of 0, negative anti-dsDNA, normal complement levels, and no proteinuria, while successfully tapering oral prednisone to 20 mg/day. This clinical course highlights the critical importance of aggressive initial immunosuppression followed by B-cell targeted biologic therapies in achieving sustained remission in severe pediatric visceral lupus. Detailed trends in laboratory parameters throughout hospitalization are summarized in Table 1. Notably, the profound hypercoagulable and inflammatory state—evidenced by a markedly elevated D-Dimer of 13.74 μg/mL and elevated CRP—resolved rapidly (D-Dimer dropped to 0.47 μg/mL, CRP normalized) following pulse therapy (Table 1).

**Table 1:** Serial laboratory findings and clinical parameters during the hospitalization.

Laboratory variables	Reference range	Day 1 (admission)	Day 4–8 (peak illness)	Pre-discharge
Hematology				
White blood cells ($\times 10^∧^9$/L)	3.5–9.5	3.9	10.9	7.8
Hemoglobin (g/L)	115–150	113	149	104
Platelets ($\times ^∧^9$/L)	125–350	142	263	194
Inflammation and coagulation				
C-reactive protein (mg/L)	< 8.0	12.95	10.5	4.33
Procalcitonin (ng/mL)	< 0.5	0.12	0.08	N/A
D-Dimer ($\mu$g/mL)	< 0.5	N/A	13.74	0.47
Chemistry and liver function				
Albumin (g/L)	40–55	38.3	32.1	32.3
Alanine Aminotransferase (ALT) (U/L)	< 40	56	40	65
Aspartate Aminotransferase (AST) (U/L)	< 35	53	48	33
Serum creatinine ($\mu$mol/L)	45–84	61.3	46.6	51.3
Immunology				
Complement C3 (g/L)	0.79–1.52	N/A	0.42	0.50
Complement C4 (g/L)	0.16–0.38	N/A	0.04	0.06
Anti-dsDNA antibody	Negative	N/A	1:10	1:32
Urinalysis				
Urine protein	Negative	N/A	++	N/A
Urine occult blood	Negative	N/A	+−	N/A
SLEDAI-2K score	0	N/A	13	N/A

WBC, white blood cell; Hb, hemoglobin; PLT, platelet; CRP, C-reactive protein; PCT, procalcitonin; ALT, alanine aminotransferase; AST, aspartate aminotransferase; C3, complement 3; C4, complement 4; Anti-dsDNA, anti-double-stranded DNA antibody; SLEDAI-2K, Systemic Lupus Erythematosus Disease Activity Index 2000. N/A: Not Available/Not Assessed.

Furthermore, transient hematuria and proteinuria (which peaked at ++ during the disease flare) completely resolved, and complement (C3/C4) levels began to recover (Table 1), objectively corroborating the remission of the visceral vasculitis storm.

Furthermore, a follow-up CECT approximately 3 weeks later confirmed significant radiological resolution of bowel wall edema (Figure 2D), perivascular exudation (Figure 2E), and ureteral wall enhancement (Figure 2F).

## 3 Discussion

### 3.1 The “Visceral Smooth Muscle Vasculitis” hypothesis

The simultaneous involvement of the gastrointestinal tract and the urinary tract in our patient supports the hypothesis of a generalized “Visceral Smooth Muscle Vasculitis/Myopathy” in SLE. The intestine and ureter are smooth, muscle-rich organs. The deposition of immune complexes (ICs) in the walls of these organs activates the complement cascade (evidenced by our patient's low C3/C4 ratio) (17), leading to inflammation, edema, and secondary smooth muscle dysfunction. While lupus enteritis and lupus cystitis are often discussed separately, our case suggests that they may represent a continuum of the same pathological processes affecting visceral smooth muscles.

### 3.2 Pathogenic mechanisms: extrinsic compression vs. intrinsic vasculitis

In clinical diagnostics, Occam's Razor (the Law of Parsimony) suggests that a single diagnosis should explain all findings. In patients with lupus enteritis who develop hydronephrosis, the prevailing theory is often “monistic”: the massive intestinal edema and ascites cause extrinsic compression of the ureters, or the inflammation extends passively to the retroperitoneum (serositis). However, our case necessitates a “dual pathology” explanation based on specific radiographic evidence:

1. Ureteral Wall Enhancement: In extrinsic compression (e.g., by a tumor or swollen bowel), the ureter is a passive victim, and its wall should remain thin and non-inflamed. Our patient's CT demonstrated clear enhancement of the ureteral walls. While this “tram-track” appearance is not pathognomonic for lupus ureteritis—as it may also be observed in infectious ureteritis, IgG4-related disease, or other systemic vasculitides (18)—it serves as a potentially useful radiologic clue. In the context of our patient's high-titer autoantibodies, low complement levels, and concurrent active enteritis, this intrinsic mural enhancement strongly suggested primary vasculitic involvement rather than mere passive secondary compression.

2. Timing of Symptoms: The patient reported dysuria *before* the peak abdominal pain. Had the hydronephrosis been purely secondary to the mass effect of intestinal edema, urinary symptoms should have appeared *after* the development of severe enteritis. The presence of dysuria suggests that the urinary tract is an independent and early target of autoimmune attacks.

### 3.3 Differential diagnosis: infection and thrombosis

Lupus enteritis often mimics surgical acute abdomen or severe infection. In our case, differentiation was complicated by the presence of fever and ascites, which initially led to a diagnosis of abdominal infection in the Nephrology department. We propose that, while secondary infection (or the risk thereof) warrants the empirical use of broad-spectrum antibiotics (Ceftriaxone, later switched to Imipenem owing to hepatotoxicity concerns), infection alone cannot explain the full clinical spectrum.

Several lines of evidence support vasculitis as a primary driver.

1. Therapeutic Response: The patient's symptoms worsened despite adequate antibiotic coverage, but resolved rapidly after high-dose steroid pulse therapy.

2. Negative Culture: Ascites fluid was exudative but culture-negative, consistent with autoimmune serositis rather than bacterial peritonitis (4).

3. Biomarker Dissociation (CRP vs. PCT): Throughout the disease course, despite the presence of fever and an elevated C-reactive protein (CRP), the patient's procalcitonin (PCT) level remained consistently normal ( ≤ 0.12 ng/mL, Table 1) (19, 20). This classic “high CRP/low PCT” dissociation is highly indicative of an autoimmune flare—specifically systemic vasculitis—rather than a severe systemic bacterial infection, providing crucial biochemical support for escalating immunosuppression instead of merely upgrading antibiotics.

4. Specific Imaging: The “Target Sign” (submucosal edema) and “Ureteral Wall Enhancement” are hallmarks of ischemic/inflammatory vessel damage, distinct from the diffuse changes seen in simple peritonitis.

5. Coagulation Profile: The excessively high D-Dimer level (13.74 μg/mL) during the peak illness strongly hinted at widespread endothelial injury and microvascular thrombosis, which is a hallmark of severe lupus vasculitis rather than localized bacterial peritonitis (21).

Furthermore, the development of splenic infarction in SLE warrants careful evaluation to differentiate between *in situ* macroscopic thrombosis driven by antiphospholipid syndrome (APS) and primary visceral vasculitis. In our patient, the comprehensive antiphospholipid antibody panel—including lupus anticoagulant (LA), anti-cardiolipin (aCL), and anti-β2-glycoprotein I antibodies—was completely negative. Consequently, we postulate that the splenic infarction was primarily mediated by severe systemic vasculitis leading to widespread endothelial injury and secondary microthrombosis, rather than a primary catastrophic APS event. Recognizing the severe hypercoagulable state, prophylactic and therapeutic anticoagulation with low-molecular-weight heparin (LMWH, 5,000 IU/day subcutaneously) was initiated promptly on Day 5 of admission, which successfully prevented further thromboembolic events.

Therefore, clinicians should recognize that in active SLE, infection may coexist with its typical pathology, but ultimately it is the visceral vasculitis itself which causes the most serious symptoms, necessitating immediate escalation of immunosuppression.

### 3.4 Therapeutic implications and prognosis

Differentiating between compression and vasculitis has profound therapeutic implications. Compression may resolve with moderate diuresis in the rest of the gut. However, lupus ureteritis carries a high risk of subsequent fibrosis and stricture formation if the inflammation is not halted rapidly (7, 8). Furthermore, ureteral wall enhancement was an alarming factor in our patient's disease progression, prompting us to escalate from low-dose steroids (40 mg) to Pulse Therapy (500 mg). The rapid resolution of the hydronephrosis in our patient validates this aggressive approach. Long-term follow-up is essential, as silent scarring can lead to obstructive nephropathy after an acute flare.

### 3.5 The neurological prodrome: a missed clue?

The patient's history of transient ptosis was relevant to her treatment. While unconfirmed, it raises the possibility of overlap syndrome (SLE + Myasthenia Gravis) or neuropsychiatric SLE (NPSLE) involving the cranial nerves. Although ptosis resolved spontaneously, its presence months before the visceral crisis suggested that the disease was active and affected the neuromuscular system (skeletal muscle) before progressing to the smooth muscle system. This highlights the protean and insidious nature of SLE onset in pediatric and adolescent patients.

### 3.6 Strengths and limitations

A major strength of our approach to this case was the timely execution of a repeat dynamic CECT during the peak of the illness, which successfully captured the fleeting but critical sign of “ureteral wall enhancement.” This prevented a misdiagnosis of simple secondary compression and guided the immediate use of high-dose pulse steroid therapy, ultimately saving the organ function. A limitation of this report is the lack of histological confirmation (e.g., intestinal or ureteral biopsy), as the patient's acute and fulminant condition, combined with severe ascites, rendered invasive biopsy procedures clinically unfeasible and ethically unjustifiable at the time.

In conclusion, this case illustrates a fulminant presentation of SLE characterized by the simultaneous involvement of the intestines, ureters, and spleen. Specifically, it underscores the limitation of relying solely on the compression theory when explaining hydronephrosis in lupus enteritis. Clinicians must actively search for ureteral wall enhancements on CT. This subtle but critical sign confirms primary Lupus Ureteritis, which mandates immediate and aggressive immunosuppression to preserve organ function. Finally, the results of this report suggest that a lower minimum criteria for mandatory dynamic imaging in patients with SLE presenting with non-specific abdominal symptoms is necessary.

## 4 Patient perspective

The patient's father shared the following perspective: “When my daughter's stomach started swelling severely and the initial antibiotics did not work, we were terrified. The turning point was when the rheumatology team took over, performed the detailed CT scan, and immediately changed the treatment to high-dose steroids and targeted medications. Seeing her abdominal pain disappear and her appetite return within just a few days was a massive relief. We are deeply grateful for the accurate diagnosis and hope her story can help other doctors recognize this rare condition earlier so other teenagers won't have to suffer as much.”

## Data Availability

The original contributions presented in the study are included in the article/supplementary material, further inquiries can be directed to the corresponding author.
